# Patient reported outcomes improve following lower limb prosthesis and socket replacement

**DOI:** 10.1186/s41687-026-01021-4

**Published:** 2026-02-21

**Authors:** Todd J. Castleberry, Patsy M. Diaz Delgado, Bretta L. Fylstra, Dwiesha L. England, Kristin E. Nalivaika, Shane R. Wurdeman

**Affiliations:** Hanger Institute for Clinical Research and Education, 10910 Domain Dr., Ste. 300, Austin, TX 78758 USA

**Keywords:** Prosthesis replacement, Socket replacement, Outcomes

## Abstract

**Background:**

Prosthetic sockets are fundamental in stabilizing the residual limb and providing a secure attachment for the prosthesis, therefore aiding in achieving a more natural and efficient walking motion. Patients who are fit with a prosthesis report increased mobility which correlates to increased quality of life and satisfaction. While timely receipt of an initial prosthesis following amputation improves patient outcomes, the impact of the socket or prosthesis being replaced is less documented. The purpose of this analysis was to assess the change in outcomes resulting from socket and prosthesis replacements.

**Methodology:**

This study included patients within a national database from a national privately owned provider of prosthetic care. Inclusion criteria consisted of adults with a unilateral, lower limb amputation that received either a replacement socket or prosthesis. Data were collected during routine clinical care with questionnaires about quality of life and satisfaction (Prosthesis Evaluation Questionnaire-Well Being), and mobility (Prosthesis Limb Users Survey of Mobility). Statistical analyses consisted of Student’s t-tests and estimated marginal means to determine significant changes within each group. Hours worn was collected as a secondary variable.

**Results:**

A total of 19,185 longitudinal outcomes were included in the final analysis and divided into four groups based on amputation level and socket or prosthesis replacement status (above knee socket replacement *n* = 2,515, below knee socket replacement *n* = 7,746, above knee replacement prosthesis *n* = 1,717, and below knee replacement prosthesis *n* = 7,207). Quality of life, satisfaction, and mobility improved significantly across all groups (*p* < 0.01), with hours worn increasing after socket replacement in both groups (*p* < 0.01) but not in the prosthesis replacement groups. Individuals with vascular disease or diabetes had reduced mobility compared to other etiologies. Older age negatively impacted mobility.

**Conclusions:**

On average, patients with lower limb amputation receiving an above-knee or below-knee socket replacement or prosthesis replacement can expect an improvement in their mobility and well-being.

**Clinical relevance:**

The findings highlight the clinical relevance of prosthetic socket and prosthesis replacements by demonstrating significant improvements in quality of life, satisfaction, and mobility for patients with lower limb amputations. This evidence supports the importance of socket and prosthesis replacements in enhancing patient outcomes and informs evidence-based clinical practices. By addressing gaps in the literature, this research provides valuable insights that can guide prosthetists and physicians in optimizing care. Overall, these results underscore the long-term benefits of replacements, advocating for prioritizing resources and support for prosthetic care to improve health outcomes and quality of life for individuals with an amputation.

## Background

For those with lower limb amputation or limb difference, the ability to walk successfully with a prosthesis can have the greatest positive impact on their quality of life compared to no prosthesis [1]. Individuals who are content with their prosthesis and find it comfortable tend to be more active, take more steps, and participate more within their community [2]. Prosthetic sockets have a strong influence over prosthesis comfort, serving as an essential component for providing a secure and stable attachment of the prosthesis to the residual limb [3]. Well-fitting sockets provide a comfortable interface to protect the person’s residual limb and promote a more natural and efficient gait. Conversely, poorly fitting sockets, can cause discomfort, skin injury, overuse injuries, and arthritis, thus affecting the person’s ability to participate in activities of daily living and social activities, resulting in decreased quality of life with potential prosthesis disuse [4, 5].

Prosthetic components beyond the socket al.so have a direct influence on prosthesis use and overall health outcomes. One example is the incorporation of microprocessor-controlled componentry. Users of microprocessor knees compared to users of non-microprocessor knees often have better functional status, mobility, and overall quality of life [6, 8] Similarly, users of microprocessor ankle-foot systems demonstrate better mobility than users of other foot technologies [9, 11]. Therefore, access to both a well-fitting socket and prosthesis with appropriate componentry is crucial for the optimal mobility and quality of life of people with lower limb amputations.

While a well-fitting socket is essential for comfort and mobility, [5], [12] its effectiveness can diminish over time due to changes in the residual limb, such as volume fluctuation or tissue maturation. These changes may compromise socket fit and, in turn, affect overall prosthesis function. Additionally, as patients progress in their rehabilitation, evolving functional needs may render their current prosthetic components inadequate. In such cases, replacing the socket alone may not be sufficient, and a full prosthesis replacement, including updated componentry may be necessary to restore optimal function and comfort.

Multiple studies have demonstrated that timely delivery of an initial prosthesis has important functional and psychosocial benefits for patients, including increased mobility, faster walking speeds, reduced dependence on assistive devices, greater community participation, reduced emergency room use, and overall improved psychological well-being [13–18]. However, there is less understanding of the effect of prosthesis componentry replacement on the health outcomes of established prosthesis users. Considering current clinical practice, where prosthesis replacements are a standard occurrence, understanding the effects of prosthesis replacements on patient outcomes is critical for informing adequate prosthesis prescription and replacement practice guidelines and policies, because these decisions directly influence long-term mobility, comfort, and quality of life. Without clear evidence, replacements may be underutilized, potentially leading to diminished function, quality of life, and reduced patient satisfaction. Evidence-based guidance ensures that replacements are provided proactively and appropriately, aligning clinical care with the evolving needs of prosthesis users.

The purpose of this study is to assess the change in quality of life, satisfaction, and mobility outcomes resulting from socket and prosthesis componentry replacements in individuals with unilateral lower limb amputations. By focusing on the impact of these replacements, the aim is to quantify the ongoing prosthetic management required to maintain or improve patient outcomes over time. We hypothesize that individuals who receive appropriate socket and componentry replacements will demonstrate significant improvements in quality of life, satisfaction, and mobility compared to their pre-replacement measures.

## Methods

### Study design

This retrospective analysis included patient reported outcomes (PROs)collected during April 2016 through June 2024 from a nationwide private prosthetic clinic system. Outcomes were collected as part of routine clinical care for adults with a unilateral lower limb amputation that received either a replacement socket or prosthesis. A replacement prosthesis was defined as replacing a prosthetic foot or knee, in addition to the socket. The pre-replacement measures were outcomes collected prior (within twelve months) to receiving a replacement socket or prosthesis and the post-replacement outcomes were collected between two weeks and twelve months following a replacement socket or prosthesis. If multiple outcome assessments were collected in these timeframes, the assessment closest to the date of delivery was used. Additionally, time since amputation, time from pre-replacement to device delivery, and time between assessments (i.e., time from pre-replacement to post-replacement) were collected as descriptive data. The (IRB name blinded for review ###) Institutional Review Board (protocol number ### blinded for review) approved the research and deemed it exempt from informed consent. The investigation adheres to the Strengthening the Reporting of Observational Studies in Epidemiology reporting guidelines for observational studies [19].

### Participants

This study included adults with unilateral lower limb amputation or limb difference transtibial and proximal. There were no exclusions based on amputation level or etiology, however, groups were identified using Healthcare Common Procedure Coding System (Centers for Medicare & Medicaid Services, 2024) L-codes for replacement sockets (L5701, L5700), and replacement prosthesis base codes (L5200, 5321, 5100, 5105, or 5301) [20]. Participants without outcomes associated with pre- and post-replacement socket or prosthesis delivery were excluded.

### Primary outcome variables

The primary outcome domains assessed were mobility, quality of life and satisfaction. Mobility was measured using the Prosthesis Limb Users Survey of Mobility (PLUS-M™) 12-item short form (v1.2) [21, 22] while quality of life and satisfaction were assessed using the Prosthesis Evaluation Questionnaire-Well Being (PEQ-WB) subsection. The PEQ-WB includes two questions evaluating satisfaction and quality of life over the preceding four weeks [23]. The instrument in its original form is administered as a continuous visual analog scale but in the current application is administered as a 10-point ordinal scale to solicit patient ratings in a clinic-friendly manner for scoring in real-time [17, 24]. The PLUS-M™ is both an instrument and key component of the broader outcomes assessment framework. The PLUS-M™ was intentionally developed and validated using rigorous qualitative and psychometric methods. Concept elicitation through focus groups ensured that items were grounded in the lived experiences of individuals with lower limb amputation, capturing relevant domains of prosthetic mobility. Subsequent application of Item Response Theory (IRT) enabled calibration of the item bank across a large, diverse population, providing several important measurement advantages. Specifically, IRT supports strong construct validity, facilitates identification and mitigation of differential item functioning (measurement invariance) across subgroups (e.g., by age, sex, etiology), and ensures that scores are comparable even when different subsets of items are administered. These features increase confidence that PLUS-M provides stable and unbiased mobility estimates across heterogeneous patient populations. The PLUS-M includes an item bank calibrated across a large and diverse population of lower limb prosthesis users [25]. The 12-item short form (v1.2) was derived from this item bank to support efficient clinical use, selecting items that span a broad range of mobility difficulty and demonstrate favorable differential item functioning [25]. Respondents rate each item on a five point scale, and the summed raw score is converted to a T-score using established calibration metrics. T-scores range from 0 to 100, with 50 representing the mean mobility level for lower limb prosthesis users. The 12-item short form yields T-scores ranging from 21.8 to 71.4, reflecting a wide spectrum of functional mobility [21].

### Statistical analysis

Descriptive data are presented as mean ± standard deviation and frequencies. Univariate paired Student’s t-tests were performed to test for significant differences in mobility, quality of life and satisfaction stratified by four subgroups from pre to post replacement timepoints (α = 0.05). The four subgroups were separately analyzed based on socket or prosthesis replacement status (above knee (AK) socket replacement, below knee (BK) socket replacement, AK prosthesis replacement, and BK prosthesis replacement). Multivariate linear mixed effect models were performed to adjust for the effect of hours worn, etiology, age, gender, time from pre-replacement to device delivery, and time between assessments. The estimated marginal means were then calculated from each mixed-effects model. Effect size was calculated with cohen’s d. All statistical analyses were evaluated using R statistical software (version 4.4.1).

## Results

**Fig. 1:**
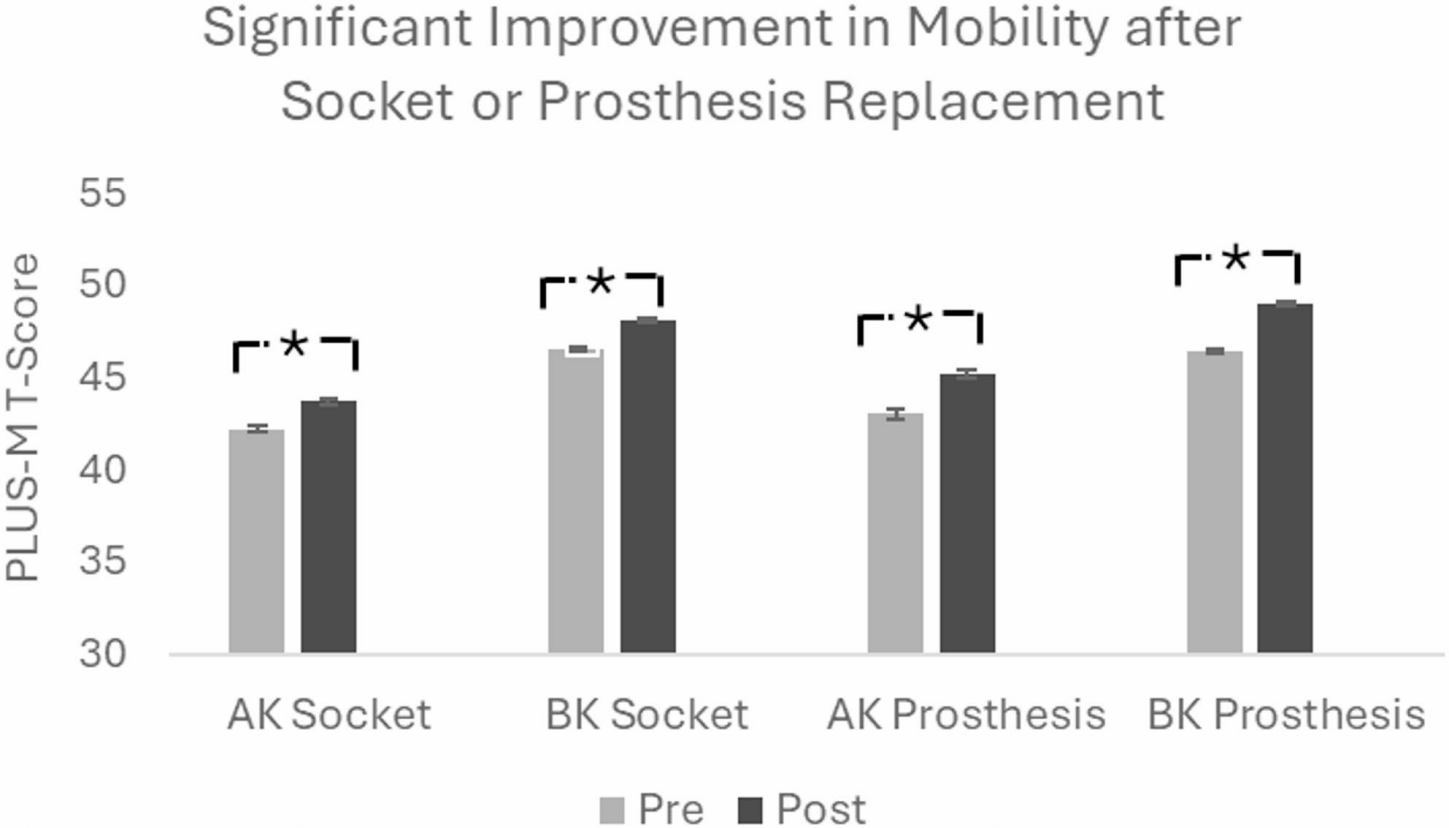
Estimated marginal means for mobility across each cohort after controlling for the effect of age, sex, etiology, hours worn, time from pre-replacement to device delivery, and time between assessments. AK=Above-Knee, BK=Below-Knee. * Represents statistical significance

**Fig. 2:**
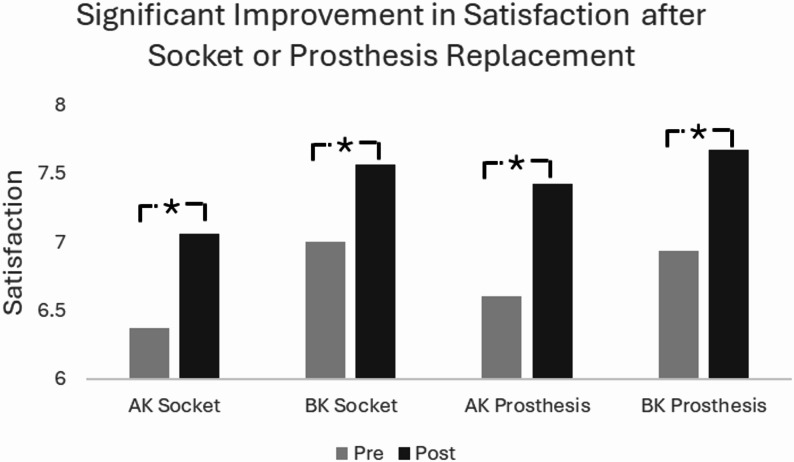
Estimated marginal means for satisfaction across each cohort after controlling for the effect of age, sex, etiology, hours worn, time from pre-replacement to device delivery, and time between assessments. AK=Above-Knee, BK=Below-Knee. * Represents statistical significance

**Fig. 3:**
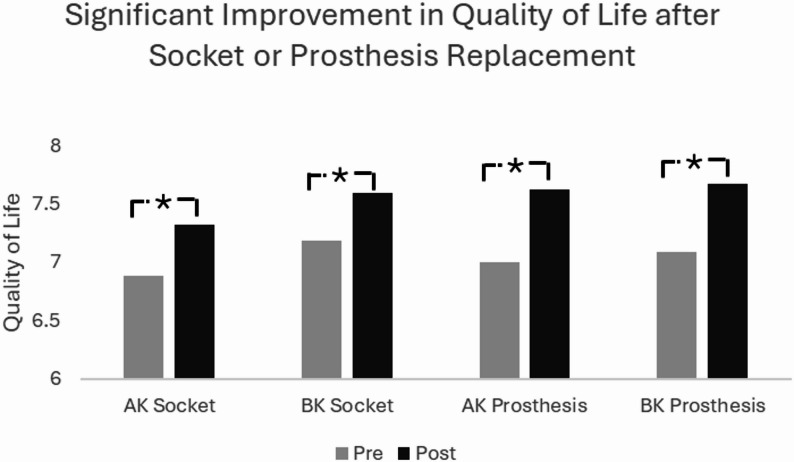
Estimated marginal means for quality of life across each cohort after controlling for the effect of age, sex, etiology, hours worn, time from pre-replacement to device delivery, and time between assessments. AK=Above-Knee, BK=Below-Knee. * Represents statistical significance

The sample included a total of 19,185 individuals with unilateral lower limb amputations who received either a replacement socket or prosthesis. The sample included 2,515 individuals receiving only an AK socket, 7,746 receiving a BK socket, 1,717 receiving an AK prosthesis, and 7,207 receiving a BK prosthesis (Table 1). The average age was 59.35 ± 13.42 years with 64.4% ≥65 years. The quality of life and satisfaction scores significantly improved following the replacement of a socket or prosthesis for all four groups (Table 1). Additionally, PLUS-M T-scores, satisfaction, and quality of life increased across all groups for socket and prosthesis replacement (Table 1; Figs. 1, 2 and 3). The effect sizes across all group for mobility, satisfaction and quality of life ranged from 0.2 to 0.3. Hours worn increased following socket replacement, but not prosthesis replacement (Table 1). Results from the multivariate analysis showed individuals with socket or prosthesis replacement had improvement in PLUS-M, quality of life, and satisfaction after controlling for age, sex, etiology, hours worn, gender, time from pre-replacement to device delivery, and time between assessments.

**Table 1:** Descriptives at pre-replacement

	AK Socket **N = 2515)**	BK Socket **N = 7746)**	**AK Prosthesis N = 1717)**	**BK Prosthesis N = 7207)**
Age (Mean ± SD)	59.50 ± 14.89	59.20 ± 12.88	57.99 ± 15.09	58.34 ± 13.37
Height (m) (Mean ± SD)	1.83 ± 4.41	1.76 ± 0.28	1.76 ± 0.82	1.90 ± 6.00
Weight (kg) (Mean ± SD)	84.70 ± 25.51	94.51 ± 27.82	84.28 ± 22.08	92.99 ± 28.77
Time Since Amputation (years) (Mean ± SD)	8.36 ± 13.08	5.20 ± 9.03	13.78 ± 16.65	8.84 ± 13.09
Time Between Pre-replacement and Follow-up (months) (Mean ± SD)	5.49 ± 4.10	5.57 ± 4.31	5.25 ± 3.73	5.26 ± 4.12
Time from Pre-replacement to Device Delivery (Mean ± SD)	3.11 ± 2.78	2.99 ± 2.86	2.87 ± 2.35	2.66 ± 2.51
Hours Worn (Mean ± SD)	8.96 ± 5.18	11.10 ± 4.62	9.91 ± 5.45	11.37 ± 4.56
Gender, n (%)				
Female	733 (29.1)	1955 (25.2)	437 (25.5)	1858 (25.8)
Male	1782 (70.9)	5791 (74.8)	1280 (74.6)	5349 (74.2)
Cause of Amputation, n (%)				
Non-dysvascular/ Non-diabetic	1162 (46.2)	2063 (26.6)	941 (54.8)	2546 (35.3)
Vascular Disease/ Diabetes	1353 (53.8)	5683 (73.4)	776 (45.2)	4661 (64.7)

**Table 2:** Unadjusted model results showing changes in outcomes after socket and prosthesis replacement

		Mobility		Satisfaction		Quality of Life		Hours Worn	
	*n*	Mean Difference [95%CI]	*P*	Mean Difference [95%CI]	*P*	Mean Difference [95%CI]	*P*	Mean Difference [95%CI]	*P*
AK Socket	2515	1.45 [1.17, 1.73]	< 0.001	0.69 [0.59, 0.79]	< 0.001	0.44 [0.35, 0.52]	< 0.001	0.41 [0.12, 0.70]	0.005
BK Socket	7746	1.57 [1.40, 1.75]	< 0.001	0.55 [0.49, 0.61]	< 0.001	0.41 [0.36, 0.46]	< 0.001	0.45 [0.31, 0.61]	< 0.001
AK Prosthesis	1717	2.11 [1.73, 2.51]	< 0.001	0.82 [0.70, 0.94]	< 0.001	0.59 [0.49, 0.70]	< 0.001	0.36 [-0.02,0.73]	0.060
BK Prosthesis	7207	2.63 [2.44, 2.83]	< 0.001	0.74 [0.69, 0.80]	< 0.001	0.58 [0.53, 0.63]	< 0.001	0.10 [-0.05, 0.26]	0.197

## Discussion

This study aimed to quantify the impact of socket and prosthesis replacements on quality of life, satisfaction, and mobility for individuals with unilateral lower limb amputation. The results demonstrated significant improvements in all primary outcomes following the replacement of sockets or prostheses, highlighting the importance of appropriate prosthetic replacements. PLUS-M T-scores are normalized to a mean of 50 (SD = 10) based on a United States reference population, which aids interpretability and benchmarking. However, interpretation outside the United States may be limited, as cultural or healthcare differences can influence mobility distributions. Although the IRT framework supports cross-cultural adaptation, further validation is needed to establish global applicability.

The significant improvements in quality of life and satisfaction scores after socket replacement observed in this analysis are consistent with previous research, indicating that well-fitting prosthetic sockets are crucial for enhancing the overall well-being of individuals with limb amputation or limb difference.[26, 27] A recent study evaluating socket comfort following a replacement socket found correlations between socket comfort and mobility utilizing the PLUS-M™ measure.[28] These findings suggests that addressing issues related to socket fit can lead to better patient outcomes, including reduced discomfort and increased participation in daily activities.[2] Additionally, results from Diment et al., 2022 suggests socket fit correlates with both steps per day and hours worn. Although the current study did not examine socket fit, there was a significant correlation between socket replacement and hours worn and mobility for the socket replacement groups (Table 1).

Notably, an increase in hours worn was observed only in the socket replacement groups, but not the prosthesis replacement groups. This distinction likely reflects underlying reasons for each intervention. Socket replacements are typically driven by issues related to poor socket fit or discomfort. These factors directly influence a patient’s ability to wear the prosthesis. In contrast, prosthesis replacements are more often prompted by issues with componentry, such as mechanical failure or a change in patient needs that require different componentry. While existing literature acknowledges a relationship between device componentry and prosthesis use, the findings of the current study suggest that socket comfort might be a more influential factor. Therefore, when a patient reports a decline in prosthesis use, it may be indicative of socket-related issues, and proceeding with a socket replacement could be a clinically appropriate and beneficial intervention. The observed correlation between hours worn and improvements in mobility, quality of life, and satisfaction further underscores the importance of addressing socket fit proactively to improve patient outcomes.

Subgroup analysis revealed that both individuals with AK and BK amputation benefited from the replacements (Table 1). While the improvements were significant, the degree of change varied between groups, particularly in terms of mobility. These differences likely stem from distinct biomechanical demands and challenges associated with each amputation level.[29] Recognizing these nuances is crucial for customizing prosthetic interventions to meet the specific needs of individuals with different levels of amputations. Previous findings indicate that mobility and quality of life scores among long-term prosthesis users remain relatively stable from initial fitting through long-term use. However, satisfaction scores tend to peak within the first 0–3 months after prosthesis receipt and then decline over time.[30] This knowledge can help assist the patients and clinicians, and other medical team members in making more informed, and shared decisions when considering replacing a socket or prosthesis.

Although not accounted for in the current study, microprocessor-controlled componentry has been shown to improve gait biomechanics and PROs compared to non-microprocessor devices. MPKs provide enhanced stability during stair descent and uneven terrain by actively managing knee flexion/extension, reducing falls and energy cost.[31, 32] These biomechanical benefits translate into higher mobility and satisfaction scores as well as greater community participation.[6, 8] Likewise, microprocessor ankles adapt to slopes and stairs, promoting smoother rollover and reduced compensatory movements, which improve comfort and confidence.[10, 33] Importantly, socket fit remains fundamental: even advanced componentry cannot achieve optimal performance if comfort and suspension are compromised. Our finding that socket replacement was closely linked to increased hours worn underscores that socket fit is a prerequisite for realizing the full benefits of technological upgrades.

The findings of this analysis underscore the value of regular outcome measurements over time in guiding clinical decision making for individuals with lower limb amputations. As the residual limb undergoes changes and the prosthesis experiences wear and tear, regular assessment of outcomes such as prosthesis use, mobility, satisfaction, and quality of life is essential for capturing the patient’s current experiences and informing necessary adjustments to ensure optimal fit and function.[34] The improvements in mobility, quality of life and satisfaction observed after socket and prosthesis replacements are clinically relevant to both patients and clinicians. While statistically significant improvements were observed, Cohen’s d analysis suggests the average benefit from socket or prosthesis replacement may be modest, yet even small gains in mobility may have meaningful practical significance for improving independence and daily function. However, this study did not employ anchor-based methods or pre-specified responder thresholds to establish individual-level clinically meaningful change. Thus, our interpretation of clinical relevance reflects group-level improvements, which have been previously shown to correlate with functional outcomes in prosthesis users. Nevertheless, these results highlight the necessity for clinicians to document changes in mobility, quality of life, and satisfaction into the notes as evidence to be used to quantify how improvements in socket fit result in real life changes for patients who rely on them every day. We recommend collecting mobility, satisfaction, and quality of life measures at a minimum every six months, during routine follow-up, or sooner if patients report discomfort, reduced wear time, or functional decline. Declines in hours worn may indicate the need for socket replacement, whereas persistent mobility limitations, decreased quality of life or satisfaction may warrant prosthesis replacement. Embedding these assessments into routine care can facilitate earlier interventions, guide shared decision-making, and help maintain long-term mobility, quality of life, and satisfaction.

In conclusion, this investigation provides valuable insights into the benefits of collecting and utilizing outcomes over time to quantify the need for socket and prosthesis replacements for individuals with unilateral lower limb amputations. The significant improvements in quality of life, satisfaction, and mobility highlight the critical role of prosthetic management in enhancing the overall well-being of individuals with lower limb amputation and limb difference. Future research should continue to explore the long-term effects of prosthetic replacements and identify strategies to optimize prosthetic care for with more specific, diverse populations such as race, gender, and amputation etiology. Future research should also explore the role of patient education and engagement in facilitating successful adoption of new prosthetic devices, as well as the longitudinal impact of replacement timing on patient outcomes. Such studies will help refine clinical guidelines and optimize patient-centered prosthetic care.

## Limitations

Some limitations should be considered when interpreting the results of this analysis. First, the prosthesis experience level and time since amputation among participants varied, which may have influenced their adaptation to a replacement socket or prosthesis. Additionally, the reason for socket or prosthesis replacement was not included in this analysis. Replacements occur for many reasons including broken componentry, changes in residual limb status, or overall health.

Variability in prosthesis experience among participants may also have influenced outcomes, as individuals newer to prosthesis use might perceive larger gains from replacement compared to more experienced users whose expectations are different. In addition, unmeasured factors such as psychological adjustment following amputation and socio-economic status (e.g., access to resources, support systems, and healthcare) likely contribute to differences in patient-reported outcomes. These factors were not captured in our dataset but warrant consideration in interpreting the findings and in designing future studies.

A key limitation of this study is that our analyses focus on outcomes relative to the pre-replacement state and do not include longitudinal trajectories beginning with initial prosthesis fitting. Evidence from the MAAT 6 study demonstrates that mobility and quality of life remain relatively stable over time, whereas satisfaction peaks shortly after prosthesis receipt and declines thereafter.[30] Our findings align with this pattern but do not capture long-term changes following replacement. Future research should examine outcomes longitudinally from the point of initial fitting through multiple replacements to better characterize the full cycle of decline and restoration. Lastly, the study did not account for componentry changes, such as transitioning from a non-microprocessor knee to a microprocessor knee as part of the replacement prosthesis, which could influence mobility outcomes. Future research should consider these factors to enhance understanding of prosthetic replacement and its impact on users.

## Data Availability

All data generated or analyzed during this study are included in this published article.
